# Case Report on Anastomosing Haemangioma: An Unusual Vascular Tumor in Kidney

**DOI:** 10.1155/2021/8847998

**Published:** 2021-01-07

**Authors:** Chun-hai Lo, Shui-ying Cheng

**Affiliations:** Department of Pathology, United Christian Hospital, New Kowloon, Hong Kong

## Abstract

Anastomosing haemangioma is a rare benign vascular neoplasm, which may mimic angiosarcoma histologically. We here present a case of anastomosing haemangioma arising from the kidney. This patient presented with a large kidney mass and adrenal mass. The clinical and radiological findings were suspicious for renal cell carcinoma with metastasis. Radical nephrectomy and adrenalectomy were thus performed. Histopathological examination and immunohistochemical studies concluded a diagnosis of anastomosing haemangioma of the kidney and concurrent adrenal cortical adenoma. It is important to differentiate this tumor from other borderline or malignant vascular neoplasms.

## 1. Introduction

Anastomosing haemangioma (AH) is a relatively new entity among soft tissue vascular neoplasms. It was first described in 2009 in the kidney and testis [1]. Since then, there are more than 60 renal cases and nonrenal cases reported in literature, all with similar histologic characteristics [2, 3]. Nonrenal cases include the testis, spermatic cord, ovary, adrenals, liver, colon, small bowel and mesentery, bladder, soft tissue, and bone [2, 4–26]. Kidney is the most frequently affected organ, while the most frequently affected site outside the kidney is soft tissue/bone [22]. Herein we describe a Chinese case of AH occurring in the kidney and give a brief review of the main differential diagnoses.

## 2. Case Presentation

**Figure 1:**
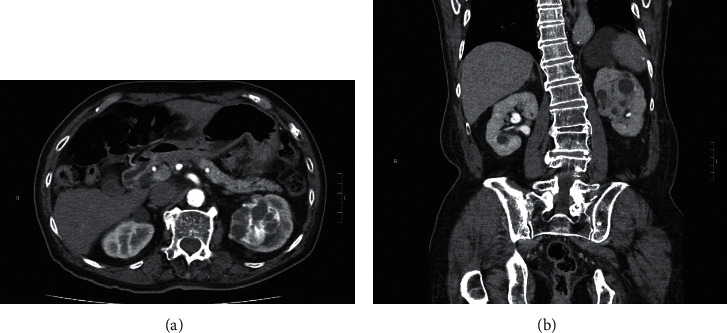
Transverse section and coronal section computer tomography imaging showing an enhancing mass lesion in the parapelvic region of the left kidney, compressing onto the renal pelvis. It has internal cystic component, tiny calcifications, and hyperdense soft tissue component.

An 84-year-old Chinese man with past medical history of hypertension, recurrent stroke, and atrial fibrillation on dabigatran presented with acute retention of urine and mild renal impairment. Light haematuria was noted after insertion of Foley catheter. The serum creatinine ranged from 130–150 *μ*mol/L. Subsequent ultrasound revealed a 5.7 cm left renal mass with cystic spaces. Computer tomography (Figure 1) showed a 5.2 cm × 5 cm × 6 cm enhancing mass lesion in the upper to mid pole of the left kidney. It had internal cystic component, tiny calcifications, and hyperdense soft tissue component. Radiological features were suggestive of cystic renal cell carcinoma.

A 2.5 cm heterogeneously enhancing soft tissue nodule was also noted in left adrenal gland. Differential diagnoses included adenoma and metastasis.

Thus, the man underwent a laparoscopic left radical nephrectomy and adrenalectomy. The surgery was uneventful. Unfortunately, the patient developed aspiration pneumonia after resuming diet and required ventilatory and inotropic support. His condition improved, and he was eventually weaned off all inotropes and ventilator around one month after operation. 2 weeks later, he developed desaturation again with fever. Chest X-ray showed bilateral haziness, left lung collapse, and effusion. His condition further deteriorated, and he eventually died of hospital-acquired pneumonia.

## 3. Pathological Findings

On gross examination, there was a light yellowish multicystic tumor in the kidney. It had a well-circumscribed and pushing border with no gross invasion into the kidney parenchyma. It measured 5.5 × 4.3 × 3 cm. There was a yellowish solid nodule in the adrenal gland. It measured 2.8 × 2.5 × 2 cm.

**Figure 2:**
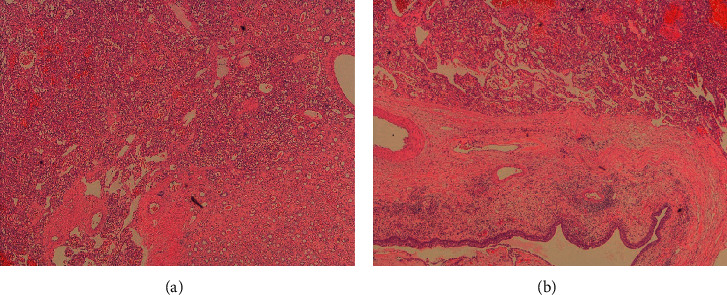
Anastomosing haemangioma with adjacent renal parenchymal tissue. The tumor is composed of splenic sinus-like vascular channels with a well-circumscribed border. No dissecting growth was noted (40×).

**Figure 3:**
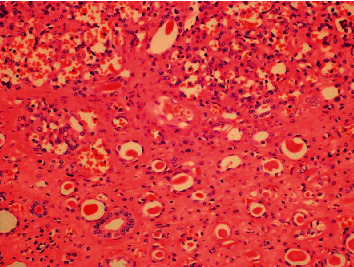
Tumor with adjacent renal tubules. The vascular channels were lined by bland endothelial cells with occasional hobnail cells (100×).

**Figure 4:**
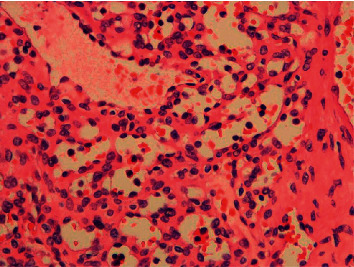
The tumor cells showed no severe cytological atypia. Mitotic activity was not detected (400×).

Microscopically (Figures 2–4), the tumor was cellular, circumscribed, and centered in the medulla of the kidney. It was comprised of anastomosing, splenic sinus-like vascular channels. The channels were lined by bland endothelial cells with occasional hobnail cells. No severe cytological atypia or mitotic activity was found. Some benign cysts were present at the periphery of the tumor.

**Figure 5:**
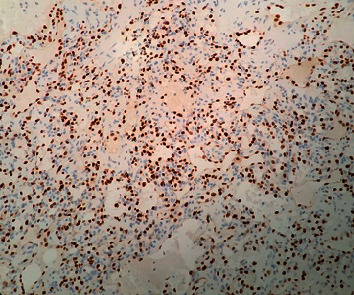
Immunohistochemical study by ERG (200×). The tumor cells show nuclear positivity.

**Figure 6:**
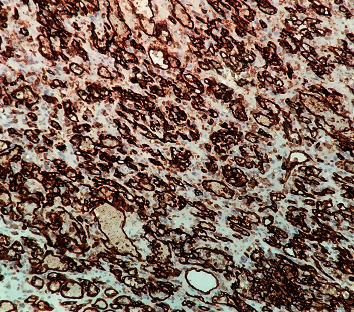
Immunohistochemical study by CD31 (200×). The tumor cells show membranous and cytoplasmic positivity.

**Figure 7:**
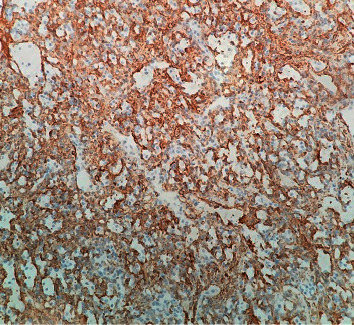
Immunohistochemical study by actin highlighting the well-developed accompanying pericytes (200×).

On immunostaining, the endothelial cells were positive for ERG and CD31 (Figures 5 and 6). Smooth muscle actin (Figure 7) highlighted the well-developed accompanying pericytes. The Ki67 proliferative index of the endothelial cells was low (<10%). The morphology together with the immunophenotypic feature was consistent with a diagnosis of anastomosing haemangioma of the kidney. Resection margin was clear.

The left adrenal nodule was an adrenal cortical adenoma featuring tumor cells arranged in compact small nests with rich capillary network in between. The cells had round nuclei, fine chromatin, eosinophilic or vacuolated cytoplasm, and normal N/C ratio. Mitotic figures were inconspicuous. There was no necrosis, cytologic atypia, vascular, or capsular invasion. On immunostaining, the cells were positive for melan-A while negative for CD10 and PAX-8. Resection margin was clear.

## 4. Discussion

This is an unusual case for coexisting adrenal cortical adenoma and anastomosing haemangioma presented at the same time. Anastomosing haemangioma is benign, and it is associated with an excellent prognosis [2, 27]. There are no reports of metastasis or recurrence after excision. Unfortunately, this patient died around 2 months after surgery due to hospital-acquired pneumonia. Among the previous case reports for anastomosing haemangioma, a significant proportion of patients was treated with total nephrectomy. It is still controversial whether total nephrectomy is necessary for such benign entity [2, 27]. However, relying on imaging may not be conclusive prior to excision. This is probably due to nonspecific imaging findings rendering an accurate preoperative diagnosis difficult [2, 27, 28].

The age of the affected patients ranges from 2 to 85 years, with median age of 49 years for renal cases and 65 years for nonrenal cases. It shows male predominance with male to female ratio of 2.3 for renal cases and 1.3 for nonrenal cases [2, 27]. The wide range of age and various sites of presentation make it hard to diagnose before the excision done.

This lesion is usually discovered incidentally during imaging studies. Less frequently, patients may present with haematuria and back pain in renal cases or mass effect and local pain in nonrenal cases [2, 27]. The most common clinical context for the development of renal AH is renal dysfunction [4, 9, 18]. Almost two-thirds of renal AH cases were reported in patients with end-stage renal disease [2, 27].

In this case, the clinical picture was complicated by the concurrent presence of an adrenal tumor. Preoperative diagnosis was thus highly suspicious for a malignant kidney tumor with metastasis.

In microscopic examination, it was obvious that the tumor is a vascular neoplasm, without any features of renal cell carcinoma. The essential histological diagnostic feature of anastomosing haemangioma is anastomosing vessels lined by hobnail endothelial cells. Desirable features include hyaline globules and extramedullary haematopoeisis [29].

The main differential diagnoses are Kaposi sarcoma and angiosarcoma [1, 2, 27, 28]. In angiosarcoma, the well-differentiated areas may also exhibit anastomosing sinusoidal-like vascular pattern and hobnail endothelial cells with limited nuclear atypia and rare mitoses. Secondly, alarming features of AH, such as lack of lobular pattern, and focal extension to larger vessels and surrounding soft tissue may be misinterpreted as signs of malignancy [1, 5, 22]. However, AH does not have a broadly infiltrative growth pattern, nor significant nuclear atypia, multilayering endothelial formations, brisk mitotic activity, high proliferative rate, or areas of necrosis. Secondly, endothelial cells of AH have a regular accompanying pericytic layer, a feature that is not observed in angiosarcoma [1]. Moreover, most angiosarcomas occurring deep in the body show high-grade histologic features or heterogeneous characteristics with well-differentiated areas admixed with poorly differentiated areas, while AHs show a homogenous pattern throughout the lesion [30].

Kaposi sarcoma is less of a concern as an increased number of plasma cells and areas of non-pleomorphic spindle cells seen in Kaposi sarcoma are not observed in anastomosing haemangioma. Kaposi sarcoma is associated with human herpes virus-8 (HHV-8) infection, and HHV-8 immunostain is highly sensitive and specific [31]. The absence of HHV-8 stain can help excluding Kaposi sarcoma.

## 5. Conclusion

Anastomosing haemangioma is a rare, benign vascular neoplasm most commonly occurring in the kidney and less commonly occurring in other body parts. The essential histological diagnostic feature of anastomosing haemangioma is anastomosing vessels lined by hobnail endothelial cells. The most important histological mimicker is angiosarcoma. The absence of significant nuclear atypia and multilayering of the endothelial cells can help making a diagnosis of AH against angiosarcoma.

## Data Availability

The case is available in United Christian Hospital intranet database, and the slides are stored in the slide storage site of the department of pathology, United Christian Hospital.
